# Body surface area is a predictor of maturity status in school children and adolescents

**DOI:** 10.1186/s12887-023-04222-8

**Published:** 2023-08-19

**Authors:** Fernando Alvear-Vasquez, Rubén Vidal-Espinoza, Rossana Gomez-Campos, Luis Felipe Castelli Correia de Campos, Evandro Lazari, Jose Francisco Guzmán-Luján, Ana Pablos-Monzó, Marco Cossio-Bolaños

**Affiliations:** 1 Universidad de Valencia, Valencia, España; 2 Universidad Católica Silva Henríquez, Santiago, Chile; 3 Universidad San Ignacio de Loyola, Lima, Peru; 4 Universidad del Bío Bío, Chillán, Chile; 5 Universidad Estadual de Campinas, Sao Paulo, Brasil; 6 Universidad Católica de Valencia, Valencia, España

**Keywords:** Maturity status, Body surface area, Children, Adolescents

## Abstract

**Background:**

Generally, Body surface area **(**BSA) changes significantly during growth and maturation. These increases portend a possible relationship between body size as determined by BSA with maturational status in children and adolescents.

**Objective:**

To determine the relationship between maturity status (MS) obtained by non-invasive anthropometric methods and body surface area (BSA) in children and adolescents of both sexes in a regional population of Chile. Additionally, we sought to verify the type of linear or nonlinear relationship between MS and BSA in both sexes.

**Methods:**

A descriptive (cross-sectional) study was designed in 950 children and adolescents of both sexes (539 males and 411 females). The age range ranged from 6.0 to 17.9 years. Anthropometric measurements were evaluated: body weight, standing height, sitting height. MS was assessed by means of two non-invasive anthropometric techniques. Both techniques predict peak years of growth velocity (APHV) through a regression equation for each sex. BSA (m^2^) was estimated by means of the Haycock equation.

**Results:**

The R^2^ in the linear model is relatively lower (R^2^ = 0.80 to 0.89 in males and 0.74 to 0.66 in females) in relation to the nonlinear quadratic model (R^2^ = 0.81 in males and 0.76 to 0.69). The quadratic nonlinear quadratic model reflected an adequate fit (RMSE) for the data set, being in men (RMSE = 1.080 and 1.125), while in women (RMSE = 1.779 and 1.479).

**Conclusion:**

BSA is positively associated with MS determined by two non-invasive methods in Chilean children and adolescents: The nonlinear quadratic model was a better fit to the data distribution. The results suggest the use of BSA as a possible predictor of maturity status in Chilean youth.

## Background

Biological maturation is defined as a progression of quantitative or qualitative changes leading from an undifferentiated or immature state to a highly organized, specialized and mature state [1]. All tissues, organs and systems of the human body mature, but do so at different times and rates [2].

In recent years, there has been widespread concern among researchers about the use of biological maturation indicators in children and adolescents in various populations around the world [3–7]. For these indicators make it possible to identify recognizable events or stages within the continuous changes that occur during the maturation process [1].

Indeed, classical indicators measuring the maturity status such as skeletal age (SA) and secondary sexual characteristics are often considered impractical and invasive [4, 5]. Thus, in recent years, non-invasive techniques have been emerging that allow predicting years from peak height velocity (a maturity compensation value). This, by using anthropometric variables, such as weight, standing height, sitting height and leg length (difference between height and sitting height) [8], and standing weight and height [9].

Both techniques have been widely used in various studies for the purpose of categorizing maturity status in children and adolescents [4, 6, 10, 11]. It is even used to verify interindividual differences in youth sports, especially during the transition to adolescence [12]. Consequently, investigating the state of maturity in the growing school population and somatic development in various regions of the world is extremely relevant.

In general, the literature highlights that traditional invasive methods bring with them logistical difficulties, limiting their uses and applications [13]. Therefore, parents, sports organizations, schools and ethics committees are often reluctant to use these protocols [12].

In this context, it is necessary to highlight new alternatives for estimating maturity status, involving the use of non-invasive somatic indicators that demand low physical and psychological risk among young people.

In fact, historically the body surface area (BSA) has been widely used to estimate body size and standardize physiological parameters since the early 20th century in adults [14, 15] and in children [16, 17]. Well, generally the BSA changes significantly in the stage of growth and maturation with the course of age, as it increases from 0.2 m^2^ at birth to 1.73 m^2^ in adulthood, along with the maturation of organ function [18].

These increases during the stage of growth and biological maturation portend a possible relationship between body size as determined by BSA with maturity status in children and adolescents. To our knowledge, no national and international studies have been found that have tested and identified non-invasive anthropometric indicators to predict maturity status during the growth and somatic development stage. Consequently, to the best of our knowledge this information could contribute to the control of interindividual variations in maturity status among schoolchildren and young athletes.

Therefore, this study aimed to determine the relationship between the maturity status obtained by non-invasive anthropometric methods with BSA in children and adolescents of both sexes from a regional population of Chile. Additionally, we sought to verify the type of linear or nonlinear relationship between MS and BSA in both sexes.

## Methods

### Type of study and sample

A descriptive (cross-sectional) study was designed in 950 children and adolescents of both sexes (539 males and 411 females). The age range ranged from 6.0 to 17.9 years. Schoolchildren were invited to participate voluntarily in the study. The participants came from 04 primary and secondary schools in the Maule region (Chile). The sample selection was non-probabilistic (quotas).

The schools that participated in the study are public and are located in the urban area of the region. One of the researchers requested permission from the school administration to collect anthropometric data.

All children and adolescents whose parents and/or guardians signed the informed consent to participate in the study and those who were within the established age range were included in the study. Students who presented locomotor problems or physical difficulties that prevented the evaluation of anthropometric measurements and those who did not attend or did not complete the anthropometric measurements were excluded.

The study was previously approved by the Ethics Committee of the Universidad (protocol no. 100/2019). The protocol was based on the Declaration of Helsinki Agreement (World Medical Association) for human subjects.

### Procedures

The data collection procedure was carried out in the facilities of each school. The Physical Education department was set up to evaluate anthropometric measurements. The data collection process was carried out in the months of October and November 2022 during school hours (8:00 a.m. to 13:00 p.m.).

Anthropometric measurements were evaluated according to the protocol described by Ross and Marfell-Jones [19]. The schoolchildren were weighed barefoot, wearing shorts and a T-shirt. A Tanita (Ltd Japan) digital scale with 100 g accuracy and a scale from 0 to 150 kg was used. Height (m) was assessed barefoot on the Frankfurt plane. An aluminum stadiometer graduated in millimeters, Seca brand, with a scale of 0–2.50 m and with an accuracy of 0.1 cm was used. The trunk-cephalic height (sitting height) was measured using a wooden bench with a height of 50 cm, with a measuring scale from 0 to 150 cm, and with an accuracy of 1 mm.

The maturity status of children and adolescents was assessed by means of the non-invasive anthropometric techniques proposed by Mirwald et al. [8] and Moore et al. [9]. The first is based on chronological age, weight, standing height, sitting height, and leg length (standing height minus sitting height) and their interactions:

The second, uses chronological age, weight and height. Both techniques predict peak years of growth velocity (APHV) through a regression equation for each sex. These techniques indicate the time before or after the APHVs.

The classification of MS was performed by means of the described by Malina, Koziel [20], classifying as average young people from − 1 to + 1 years of PHV, those younger than − 1 years of PHV classified as early and those older than + 1 years of PHV classified as late. This classification was applied to the two techniques that estimate MS (Mirwald and Moore).

The BSA (m^2^) was estimated by means of the equation of Haycock et al. [21] This equation was proposed in infants, children and adults from height (length) and weight and their corresponding allometric adjustments: BSA = 0.024265×weight (kg)0.5378 × height (cm) 0.3964.

The quality control of the anthropometric measurements was determined by the relative technical measurement error TEM (intra-evaluator and inter-evaluator). It was evaluated at 10% of the total sample. For both cases, the values ranged from 0.8 to 1.2%.

### Statistical analysis

The normal distribution of the data was verified by the Kolmogorov-Smirnov test. Descriptive statistical analysis of arithmetic mean, standard deviation and range was performed. Differences between genders were determined by Student’s test for related samples.

Regressions by sex and for each MS prediction method were performed using the linear and quadratic model. The coefficient of determination R^2^ and root mean square error (RMSE) were calculated. The MS of Mirwald et al. [8] and Moore et al. [9] were considered as dependent variables, while BSA, as independent: for Males [SM-Mirwald = -11.297 + 6. 594*BSA (Linear model), SM-Mirwald = -16.792 + 14.418*BSA-2.636*BSA^2^ (Quadratic model)]; Females [SM-Mirwald = -14.462 + 11.226*BSA (Linear model), SM-Mirwald = -23.905 + 24.909*BSA-4.766*BSA^2^ (Quadratic model)].

Males [SM-Moore = -10.694 + 6.810*BSA (Linear model), SM-Moore = -17.098 + 15.929*BSA-3.072*BSA^2^ (Quadratic model)]; Females [SM-Moore = -10.525 + 7.767*BSA (Linear model), SM-Moore = -20.984 + 22.918*BSA-5.278*BSA^2^ (Quadratic model)].

In all cases, a p < 0.05 was considered significant. Calculations were performed in Excel spreadsheets, SPSS 18.0 and R.

## Results

The anthropometric variables characterizing the sample studied can be seen in Table 1. The MS categories were determined by two methods (Mirwald and Moore). When age and anthropometric variables were compared by Mirwald and Moore, there were significant differences between both methods in the pre-pubertal category and in the pubertal category (p < 0.05). However, there were no differences between both methods in the post-pubertal category (p > 0.05).

In females, there were significant differences between both methods in the pubertal category (p < 0.01), however, in the other categories such as pre-pubertal and post-pubertal, there were no significant differences (p > 0.05).

In general, schoolchildren of both sexes categorized as pre-pubertal, evidenced lower age and lower anthropometric parameters in relation to those categorized as pubertal and post-pubertal (p < 0.05), even, pubertal presented lower values in relation to post-pubertal (p < 0.05).

**Table 1:** Anthropometric characteristics of children and adolescents categorized by maturity status

MS		Age (years)		Weight (kg)		Height (cm)		SH (cm)		LL (cm)		BSA (m^2^)	
	n	X	SD	X	SD	X	SD	X	SD	X	SD	X	SD
Categories	MS males (Mirwald)												
Pre Puberty	295	10.7	2.6	41.6	13.1	142.8	15.3	74.2	7.2	68.6	9.6	1.27	0.25
Puber	70	14.8a	0.7	62.8a	10.8	167.3a	5.4	86.9a	2.6	80.4a	5.5	1.70a	0.14
Post Puber	174	16.8a.b	0.8	70.3a.b	12.2	171.4a.b	6.4	90.4a.b	3.3	81.0a.b	5.8	1.82a.b	0.15
Total	539	13.2	3.4	53.6	18.3	155.2	18.2	81.1	9.6	74.2	10.1	1.5	0.33
	MS Males (Moore)												
Pre Puberty	188	9.2*	1.9	35.4*	10.3	133.8*	10.8	70.4*	5.7	63.4*	7.1	1.13*	0.19
Puber	177	13.9†	1.1	56.5†	11.7	161.4†	7.2	83.8†	5	77.6†	5.5	1.58†	0.16
Post Puber	174	16.8	0.8	70.3	12.2	172.1	5.6	89.8	3.9	82.3	5.2	1.82	0.15
Total	539	13.2	3.4	53.6	18.3	155.2	18.2	81.1	9.6	74.2	10.1	1.5	0.33
	MS Females (Mirwald)												
Pre Puberty	120	8.8	1.5	34.3	7.9	132.8	9.8	69.3	4.7	63.5	5.9	1.11	0.16
Puber	29	11.2a	0.6	46.7a	8.8	149.8a	4.5	77.7a	3.7	72.0a	3.7	1.39a	0.12
Post Puber	262	15.4 a.b	2	59.0a.b	12	157.6a.b	5.5	83.2a.b	4.1	74.4a.b	4.7	1.59a.b	0.15
Total	411	13.1	3.5	50.9	15.5	149.8	13.1	78.8	7.6	71.1	7	1.43	0.26
	MS Females (Moore)												
Pre Puber	101	8.5	1.4	32.8	7.2	130.4	8.5	68.2	4.2	62.2	5.2	1.08	0.14
Puber	117	11.9†	1.1	50.8†	9.8	152.9†	6.6	79.5†	4.2	73.4†	4.2	1.46†	0.15
Post Puber	193	16.3	1.2	60.5	12.6	158.2	5.4	83.8	4.2	74.3	4.9	1.61	0.16
Total	411	13.1	3.5	50.9	15.5	149.8	13.1	78.8	7.6	71.1	7	1.43	0.26

Legend: MS: maturity stage, SH: Sitting height, LL: leg length, BSA: body surface area, x: mean, SD: standard deviation, *: significant difference between pre-pubertal categories, †: significant difference between pubertal categories

**Fig. 1:**
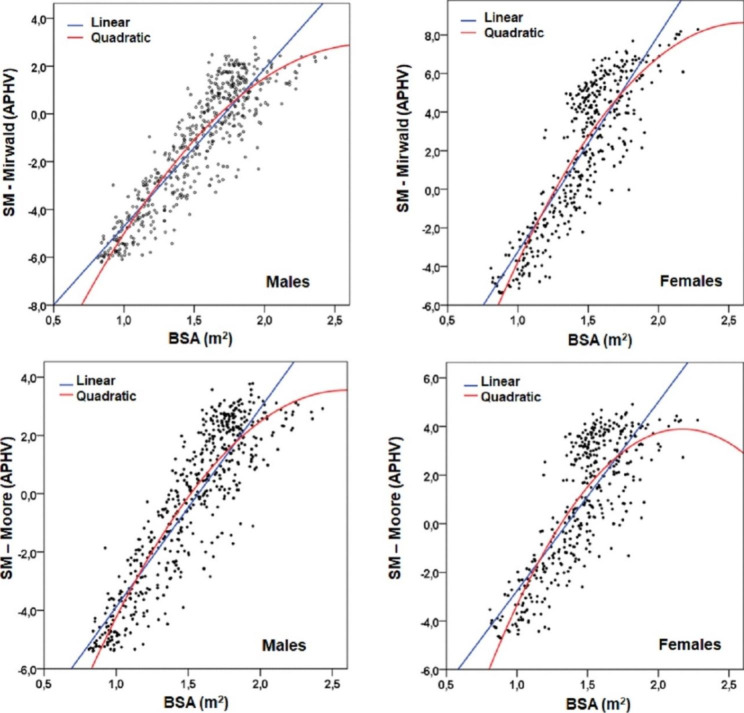
Non-linear and non-linear relationship between BSA values and MS determined by two non-invasive techniques

The linear and nonlinear regression values between MS with BSA can be seen in Table 2. The R^2^ in the linear model is relatively lower than the nonlinear (quadratic) model. The quadratic model showed a higher explanatory power from 1 to 3%. In general, the nonlinear quadratic model reflected an adequate fit (RMSE) for the data set, being in males (RMSE = 1.080 and 1.125), while in females (RMSE = 1.779 and 1.479). Figure 1 shows a parabola product of the cubic model, where pubescent males (-1APHV to + 1PHV) evidenced a BSA from 1.55 to 1.79m2 by Mirwald technique and 1.57 to 1.79m2 by Moore technique, while pubescent females (-1APHV to + 1PHV) reflected a BSA from 1.32 to 1.45m2 by Mirwald and 1.36 to 1.58m2 by Moore.

**Table 2:** Linear and non-linear regression models between MS by both methods with BSA in children and adolescents of both sexes

Models	MS (Mirwald et al 2002)					MS (Moore et al 2015)						
	R^2^	F	g1	g2	p	RMSE	R^2^	F	g1	g2	p	RMSE
Males												
Linear	0.8	2147.8	1	537	0.000	1.13	0.79	2063.5	1	537	0.000	1.19
Quadratic	0.81	1206.7	2	536	0.000	1.08	0.81	1193.2	2	536	0.000	1.125
Females												
Linear	0.74	1186.9	1	409	0.000	1.84	0.66	778.1	1	409	0.000	1.57
Quadratic	0.76	653.9	2	408	0.000	1.779	0.69	469.1	2	402	0.000	1.479

Legend: MS: Maturity stage, RMSE: root mean square error

## Discussion

The results of the study have shown that there is a linear and non-linear relationship between MS and BSA in both sexes. However, the best fit in data concentration was observed in the quadratic non-linear relationship, where the explanatory power was higher from 1 to 3% and the RMSE evidenced lower values (RSME = 1080 and 1.175 in males and RMSE = 1.779 and 1.479 in females), with respect to the linear model (RSME = 1.130 and 1.190 in males and RMSE = 1.740 and 1.570 in females).

These findings indicate that the BSA can be considered an excellent predictor of MS in children and adolescents in a regional population of Chile. Therefore, future studies that intend to propose new methods for the evaluation of MS by anthropometry could include BSA in their prediction models.

In fact, since the publication of the study by Mirwald et al. [8], in which the SM prediction equation is proposed in both sexes, more than 20 years have elapsed, in which it has been widely used in school [5, 22, 23] and sports populations [24–26]. Even, the same study group using the same database, made an adjustment to the initial equations, in which seated height was eliminated, and published new equations with a lower prediction error (known as Moore equations).

In this study we verified similar explanatory power (quadratic relationship) between BSC with the Mirwald and Moore Eq. (81%). However, in women, the explanatory power was higher with the Mirwald Eq. (79%) and lower with the Moore Eq. (69%). These findings indicate that it appears that the Mirwald and Moore technique may be appropriate methods for estimating MS in men, whereas in women only the Mirwald technique is appropriate.

In fact, the few studies carried out on this subject generally vary in their estimates from fair to moderate between methods [27, 28]. Therefore, given the absence of a criterion method to compare SM estimates, it is necessary that future studies plan a large-scale longitudinal investigation to clarify these gaps and discrepancies between methods.

In general, the original Mirwald equation has been criticized for its predictive ability, in which several limitations are highlighted in both sexes [4, 20, 29]. Even, recently some studies highlight that the equation modified by Moore et al. [9] presents some important limitations that should be considered by researchers [4, 20, 28].

Therefore, perhaps it could be timely to include BSA in these two techniques predicting MS in children and adolescents of both sexes. In addition, the nonlinear relationship in both techniques should be considered, which could improve the explanatory power in the prediction models.

In essence, body size and body build over the years has been changing in pediatric patients of ethnic/racial background [30–32], thus equations predicting MS possibly require updating their regression models.

For it is widely known that the techniques used to predict MS need periodic evaluations, so BSA could be an appropriate predictor to control for such variations in body shape and build during the growth and development stage.

In summary, although BSA is related to both techniques of MS in children and adolescents of both sexes, classically BSA has been considered by Brozek et al. [34] as a long-established measure in measurements such as overlay, triangulation, planimetry, prediction from major body dimensions. Therefore, attention should be paid as a possible predictor of MS, as it is not surprising that several expressions correlating body surface area with direct measurements of body mass and length have been reported in the literature [35]. Although, additional or alternative variables and methods need to be considered as a complement to estimate somatic maturation [30].

In fact, maturation is a complex process involving changes in body structure and function, and these changes may vary among individuals and populations. Thus, their assessment by anthropometric techniques can help predict the maturity shift and accommodate individual differences among adolescents [8].

Meanwhile, studying methodologies to assess biological maturation during the stage of growth and development remains a gap, so future studies should continue to address strategies to generate new noninvasive methodologies.

Overall, this study has some limitations in its cross-sectional design, so future studies should use longitudinal designs to confirm our findings. For example, the most commonly used indicator of maturity in longitudinal studies during adolescence is peak PHV growth velocity which require serial measurements over several years around the years surrounding the onset of peak growth velocity [36]. In addition, we also highlight that it was not possible to model these data for children and adolescents of both sexes through mathematical models such as Preece-Baines [37] and Sitar [38]. It is suggested that future studies analyze such models and verify the associations with PHV. However, notwithstanding the above, we emphasize that this study is one of the first investigations that demonstrated the usefulness of BSA in children and adolescents. Furthermore, this information may be useful for researchers who have longitudinal databases, in which the use and applicability of the BSA in children and adolescents from various geographic regions of the world can be tested.

## Conclusion

This study concludes that the BSA is positively associated with MS determined by two non-invasive methods in Chilean children and adolescents: The nonlinear quadratic model best fit the data distribution. The results suggest the potential use of the BSA as a possible predictor of MS in young athletes and non-athletes and open new possibilities and alternatives in biological maturation research to explore its validity and reliability.

## Data Availability

The datasets supporting the conclusions of this research article are available by emailing the corresponding author.

## Abbreviations

BSA: Body surface area
MS: maturity status
APHV: peak years of growth velocity
TEM: technical measurement error
RMSE: root mean square error
